# Effects of positive education intervention on growth mindset and resilience among boarding middle school adolescents in China: a randomized controlled trial

**DOI:** 10.3389/fpsyg.2024.1446260

**Published:** 2024-11-25

**Authors:** Gao Jianping, Samsilah Roslan, Kim Geok Soh, Zeinab Zaremohzzabieh

**Affiliations:** ^1^Faculty of Educational Studies, Universiti Putra Malaysia, Serdang, Selangor, Malaysia; ^2^Women and Family Studies Research Center, University of Religions and Denominations, Qom, Iran

**Keywords:** adolescents, boarding school, growth mindset, intervention, positive education resilience

## Abstract

**Introduction:**

The impact of a growth mindset and resilience on boarding middle school adolescents has received increasing attention from scholars. Nevertheless, research on how to intervene in the growth mindset, and resilience of boarding school adolescents needs further verification. The purpose of this study is to explore whether positive education intervention based on the PERMA (positive emotions, engagement, relationships, meaning, and achievement) model will help Chinese boarding middle school adolescents improve their growth mindset and resilience.

**Methods:**

This study is a randomized controlled trial with both a control group and an experimental group, including pre-tests and post-tests. The study subjects were 167 adolescents, including 84 (*n*_1_ = 84) adolescents in the experimental group and 83 (*n*_2_ = 83) adolescents in the control group.

**Results:**

The results showed that adolescents under the intervention condition reported significantly improved growth mindset and resilience.

**Discussion:**

Compared with the control group, resilience significantly increased. These findings indicate that positive educational intervention is a promising approach to improve boarding adolescents ‘growth mindset and resilience.

## 1 Introduction

Boarding schools in China have a development history of more than 60 years (Yang, 2024). By 2020, the number of rural boarders has exceeded 32 million, including 9.346 million primary school boarders (Tang et al., 2020). Boarding adolescents in China may face many stresses and challenges, such as being separated from their families, adapting to a new environment, coping with academic and social pressure, etc. (Wang et al., 2017). However, middle school is a critical stage in the educational journey of adolescents, which is characterized by cognitive, emotional, and social changes (Strahan and Poteat, 2022). Boarding middle school adolescents face unique experiences that require resilience and adaptive coping strategies during the challenges of this developmental period (Gao et al., 2021). Positive education programs are based on school-based well-being interventions that combine traditional academic curricula with empirical techniques to improve adolescents’ well-being (Seligman et al., 2009). Chinese researchers have been interested in positive psychology for nearly two decades, but their research on growth mindset and resilience has just begun (Zhang et al., 2022). Sun et al. (2021) investigated the psychological problems of middle school adolescents in all-boarding schools. The results showed that the detection rate of mild mental health problems was 47.77%, the detection rate of moderate mental health problems was 9.70%, and the detection rate of severe mental health problems was 0.37%. Boarding school adolescents often lack focus on developing a growth mindset and resilience. These collective challenges suggest a potential risk to the well-being of middle school adolescents. Further research is essential to enhance our understanding of the obstacles and supports to well-being within this population.

A growth mindset, a concept pioneered by psychologist Dweck, emphasizes the belief that intelligence and abilities can be cultivated through dedication, learning, and perseverance (Dweck, 2006). Adolescents with a growth mindset believe that intelligence is moldable and constantly changing (Zeng et al., 2016). Young people with a developmental mindset tend to see failure as an opportunity to learn and challenges as something that makes them stronger (Ortiz Alvarado et al., 2019). Resilience refers to encountering changes, challenges, and setbacks; persistence, responsive coping and recovery after disappointment, adversity, or adversity, and making it return to a reasonable level of happiness (Noble and McGrath, 2012). In the context of education, fostering a growth mindset is associated with improved academic performance, motivation, and overall psychological resilience (Yeager and Dweck, 2012). Resilience, in turn, refers to an individual’s capacity to rebound from setbacks, adapt to challenges, and maintain a positive trajectory despite adversity (Sisto et al., 2019). A growth mindset and resilience are both integral components of students, impacting academic success and long-term well-being (Skinner et al., 2020). Boarding schools, with their distinctive environment and immersive communal living, offer a unique setting to explore the impact of educational interventions on students’ mindset and resilience (Benveniste, 2018).

Positive education serves as a framework that integrates academic learning with the promotion of character strengths, well-being, and social–emotional skills. Seligman and Csikszentmihalyi (2000) note that positive psychology research is built on three main pillars: “positive subjective experiences, positive personal traits, and positive institutions.” In recent years, educators and researchers have become increasingly interested in incorporating positive educational interventions into traditional curricula to create more balanced and enriched educational experiences (Alam, 2022). According to Kern et al. (2015), Seligman’s PERMA model is a good basis for guiding the use of Positive Education in educational settings. PERMA essentially represents positive emotions, engagement, relationships, meaning, and accomplishment (Seligman, 2018). The combination of mental health education content and positive psychology concepts has greatly expanded the content and form of mental health education in universities, middle schools, and primary schools. Positive education helps promote adolescents’ growth mindset by cultivating students’ positive emotions, resilience, self-efficacy, and self-mastery (Norrish et al., 2013). For example, positive education can help adolescents better understand their strengths and challenges and view challenges as opportunities rather than obstacles (Seligman et al., 2009). At the same time, positive education can also help adolescents better cope with setbacks and failures, thereby enhancing their resilience and self-efficacy (Green et al., 2021).

To date, despite the exploration of the benefits of positive education in various contexts, much of the work has been conducted in higher education settings. As a result, research findings and conclusions are susceptible to the influence of school and adolescent characteristics, limiting the generalizability to other environments. Furthermore, existing research still lacks investigations into the impact of a positive education intervention on the growth mindset and resilience of boarding secondary school adolescents. This study aims to address this gap by rigorously examining the effects of a positive education intervention on developing a growth mindset and resilience in boarding secondary school adolescents. It provides evidence-based strategies to support adolescents’ holistic development within the unique context of boarding schools.

## 2 Literature review

### 2.1 Chinese boarding adolescents

In China, boarding school environments can create a sense of community and shared goals among adolescents (Zhu, 2019). Positive peer influence and collaboration can contribute to the development of a growth mindset, as adolescents may support each other in facing challenges and learning from mistakes. With the increase in boarding schools, there is more and more research on adolescents in boarding schools. Because boarding adolescents are used to being out of the guidance of their parents, they will have more emotional fluctuations and face various mental and physical problems (Kim et al., 2015). Zhou et al. (2009) studied the mental health status of 150 boarding middle school adolescents and found that 44% had psychological problems. Zhao and Chen (2016) found that the mental health status of non-boarding adolescents was significantly better than that of boarding students, especially the life satisfaction and negative emotions of boarding adolescents were significantly worse than those of non-boarding students. Zhi and Feng (2014) research shows that boarders have more interpersonal problems than non-boarders. Although the above research has proposed and confirmed the psychological problems of boarding survival, no research has proposed effective interventions and positive coping strategies.

### 2.2 Growth mindset

A growth mindset is defined as an individual’s implicit cognitive belief about the variable degree of his or her basic traits (e.g., intelligence, ability) (Dweck, 2006). Dweck (2017) further based on his previous research, that a growth mindset is a plastic implicit thinking pattern pointing to individual traits (such as intelligence). A growth mindset is all about working hard, accepting challenges, learning from adversity and failure, being gritty, having a positive attitude, having a subtle way of acquiring personality and maintaining a mindset that equals bringing out your best performances and stereotypes. Zeng et al. (2016) found that a mindset intervention for junior and junior high school students improves adolescents’ psychological well-being and school engagement through enhancing resilience. Zeng et al. (2016) believes that a growth mindset is a positive psychological quality that needs to focus on cultivating and improving adolescents’ growth mindset in education. Mindset theory is often applied in the context of learning and education (Boyd, 2015; Asbury et al., 2016). Adolescents with a growth mindset are more resistant to negative events (Wang and Amemiya, 2019).

In China, scholars have also done much research on the growth mindset. Some studies focus on adolescents’ intellectual plasticity (Joo et al., 2023). And improving academic performance, some studies focus on the training and development of growth mindset courses for teachers and parents (Cheng et al., 2017; Seaton, 2018). However, the research should not only be limited to this but should improve it and explore newer, more effective, and longer-lasting intervention programs on this basis for boarding school students.

### 2.3 Resilience

Resilience, as described by Sisto et al. (2019), is “a dynamic process encompassing positive adaptation within the context of significant adversity.” This definition underscores that resilience is not a fixed trait but rather a fluid process through which individuals respond to challenges and emerge with adaptive strengths. It is conceptualized through three main lenses: resilience as an outcome, a personality trait, and a developmental process (Manjula and Srivastava, 2022). These frameworks highlight that resilience can be cultivated over time, evolving through interactions with environmental and personal factors. In the context of adolescents in boarding schools, resilience takes on particular significance due to the unique adversities they face, such as separation from family and the pressures of adjusting to a highly structured and isolated environment. These challenges may increase their vulnerability to stress, making resilience a vital component of their mental and emotional well-being.

Despite existing research demonstrating the effectiveness of resilience interventions, studies have primarily focused on younger children in primary school settings (Wang et al., 2021). Adolescents in boarding schools, however, face distinct developmental and emotional challenges that necessitate more targeted intervention efforts. While psychological resilience interventions have shown promise in enhancing coping skills and emotional regulation in students, there remains a gap in the literature concerning adolescents in boarding environments. This underscores the importance of developing and evaluating interventions, such as those grounded in positive education, that specifically address the resilience-building needs of middle school students in these settings. Filling this gap could contribute to better supporting the mental health and adaptation processes of these adolescents.

### 2.4 Positive education

Positive psychology research and theory provide the general public with numerous methods to improve happiness and address issues like depression and anxiety disorders. In recent years, the concept of positive education has emerged, integrating principles of positive psychology into educational settings (Green et al., 2021). The primary goal of positive education is to promote social prosperity and enhance mental health within schools (Trask-Kerr et al., 2023). Researchers and practitioners in positive education advocate for the idea that schools can effectively “teach the skills and techniques of happiness” (Alam, 2022). According to the Global Happiness Council (2018), over 10,000 schools in China have adopted positive education practices.

Positive education provides access to a curriculum that emphasizes guiding participants toward positive experiences and leveraging individual strengths (Kern and Wehmeyer, 2021). A recent study has called for further research to evaluate the effectiveness of positive education interventions across diverse contexts (Schiavon et al., 2020). Integrating positive education interventions within a framework that emphasizes positive emotions, engagement, relationships, meaning, and achievement offers a holistic approach to fostering a growth mindset and resilience among adolescents in boarding schools. Positive education aims to cultivate well-being and psychological health in educational environments (Green et al., 2021; Trask-Kerr et al., 2023). Unlike traditional educational models, positive education focuses on nurturing positive experiences and recognizing individual strengths, thereby enhancing students’ well-being and academic performance (Kern and Wehmeyer, 2021).

The framework aligns with the goals of positive education by promoting well-being through the development of intrinsic motivation and engagement. When students are empowered to make choices and pursue activities that resonate with their personal values, they are more likely to engage deeply in their learning experiences and find meaning in their education. This intrinsic motivation fosters a growth mindset, where challenges are perceived as opportunities for self-improvement. Additionally, the emphasis on achievement allows students to experience mastery through their efforts, which reinforces their resilience. Furthermore, supportive relationships are essential in helping students navigate challenges and maintain emotional well-being, particularly in the social context of boarding schools. By adopting a positive education framework, educational settings not only enhance well-being but also cultivate the resilience and growth mindset necessary for long-term success and adaptability in both personal and academic domains.

### 2.5 Present study

Due to the late start of positive psychology research in my country, most practical projects still tend to draw on foreign research results as a whole, and lack sustainable projects that comprehensively promote positive education to the entire school; or combine it with the school environment. It is not very close, and there are even frequent conflicts between active education experiments and the school’s so-called mainstream teaching methods/content. Because of these current situations, it is very important and urgent to systematically think about the practice of positive education in Chinese schools in the context of schools, combined with the development trends, experiences, and results of international positive psychology and positive education (Xie et al., 2023).

In the Chinese educational context, this study aims to incorporate positive education intervention into boarding middle schools. According to the literature, no previous research has been conducted in Chinese boarding middle schools to implement interventions to promote a growth mindset and resilience among middle school students. Research shows that intervention research is critical to understanding the complex links between positive education and student resilience. For example, Brunwasser et al. (2009) found that many intervention programs, such as the Penn Resiliency Program (PRP), improved students’ overall well-being. To the researchers’ knowledge, this is the first study to examine the impact of a positive education intervention on increasing growth mindset and resilience among adolescents in Chinese boarding schools. The specific purpose of this study was to investigate the potential for positive educational interventions to help promote a growth mindset and resilience among middle school students. Therefore, to achieve the above goals, the following research questions are posed:

- Does the effectiveness of positive education intervention improve the growth mindset of boarding middle school adolescents in China?
- Does the effectiveness of positive education intervention improve the resilience of boarding school adolescents in China?

## 3 Methodology

### 3.1 Design

This study employed a randomized controlled trial (RCT) with two repeated measures (pre-test and post-test). Participants were randomly assigned to either the experimental group or the control group through a randomized controlled group trial.

### 3.2 Participants

The target population consisted of boarding school adolescents in Yuci District, Jinzhong City, Shanxi Province, China. Inclusion criteria for participation were carefully defined to ensure the study focused on a specific demographic group that could provide meaningful insights into the effects of the intervention. Eligible participants were required to be (1) between 12 and 16 years old, which corresponds to the developmental stage where adolescents are particularly susceptible to interventions aimed at enhancing resilience and mindset, (2) currently enrolled in a boarding school to ensure a consistent educational environment, (3) able to provide informed written consent from their parents or legal guardians, emphasizing the ethical consideration of involving minors in research, and (4) able to complete both pre-and post-test assessments were included in the final analysis.

**Figure 1:**
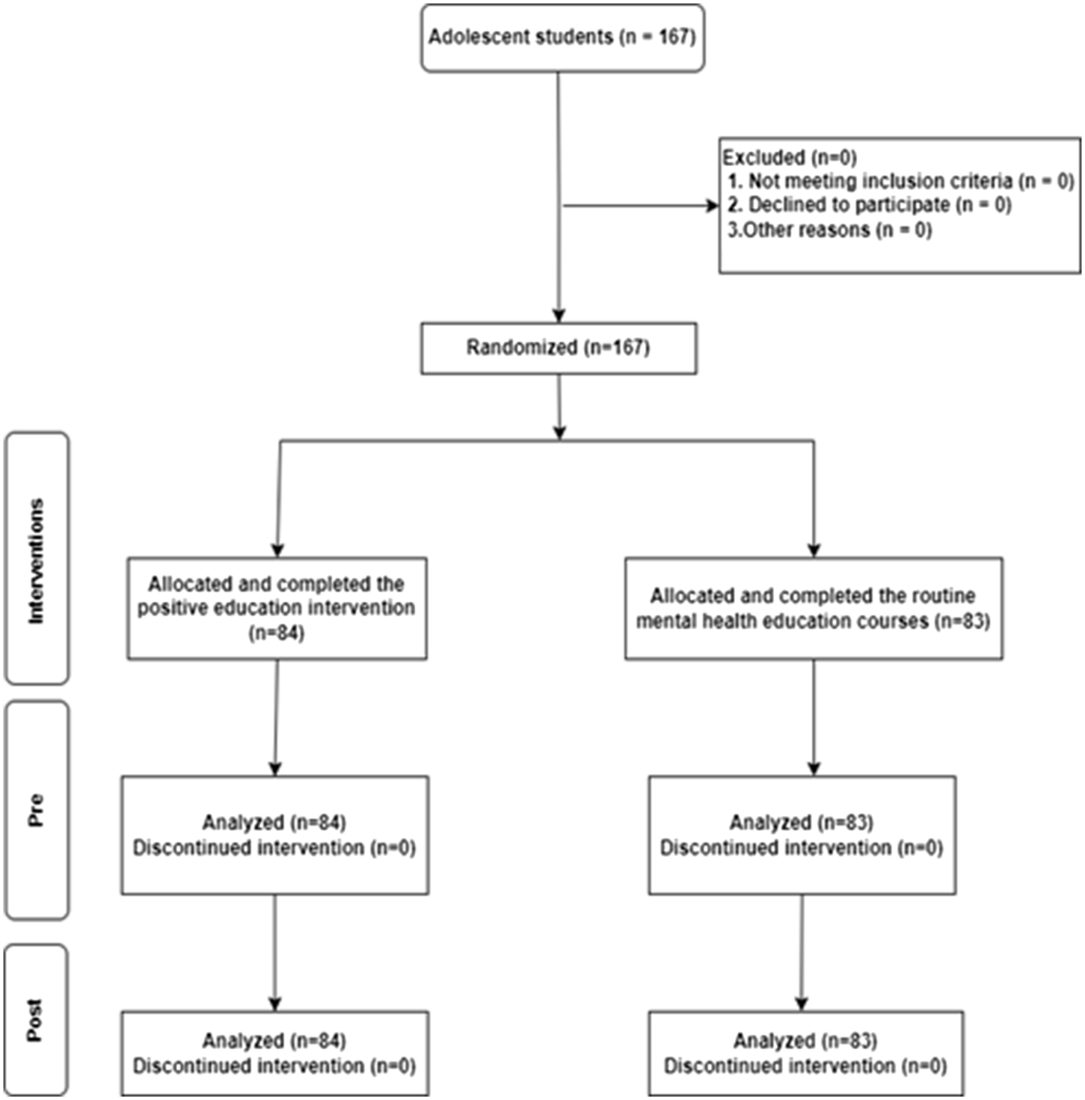
Flow diagram of the recruitment process.

Exclusion criteria were also established to maintain the integrity of the study’s findings. Participants were excluded if they (1) attended fewer than 80% of the intervention sessions (less than 12 sessions), which could impact the potential benefits derived from the program, (2) received disciplinary sanctions from the school, as such sanctions may affect their psychological state and engagement with the intervention, and (3) did not obtain informed written consent from their parents or guardians, highlighting the importance of parental involvement in adolescent research. The final sample size included 167 adolescents, with 84 students assigned to the experimental group and 83 to the control group. Participant flow is displayed below (refer to Figure 1).

### 3.3 Procedure

Participants were recruited using a multistage random sampling method. Stratified cluster random sampling based on grade levels (Grades 7, 8, and 9) was used. Six classes were randomly selected using the fishbowl technique to ensure representation and reduce selection bias. Students and their parents or guardians were informed about the study’s objectives, and written consent was obtained.

After recruitment, participants were randomly assigned to either the experimental or control group. Both groups completed pre-test assessments before the intervention. The intervention was conducted in classrooms over 12 weeks, from September 2023 to November 2023. Post-test assessments were collected after the intervention concluded.

The study received approval from the Human Research Ethics Committee of Universiti Putra Malaysia (Ref. no: JKEUPM-2023-026). Participation was voluntary, and participants could withdraw at any time without providing reasons. Informed consent was collected from both students and their parents or guardians.

### 3.4 Intervention

Participants in the experimental group underwent a 12-week positive education intervention aimed at enhancing growth mindset and resilience. The intervention was based on the PERMA model (Seligman et al., 2009), which includes five key components: positive emotions, engagement, relationships, meaning, and accomplishment. Each week, participants engaged in 60-min sessions that comprised mindfulness meditation, instructional videos, practical activities, and reflective writing exercises. These sessions were designed to foster emotional regulation and well-being, as well as the active application of growth mindset strategies. In contrast, participants in the control group attended their routine mental health education courses, which were conducted in accordance with the Special Action Plan to Comprehensively Strengthen and Improve Students’ Mental Health in the New Era (2023–2025). The control group received traditional, lecture-based instruction on mental health topics. Both groups received the same amount of instructional time to ensure consistency in exposure.

The design of the positive education intervention draws inspiration from Galante et al.’s (2021) eight-week program, which focuses on enhancing mental and emotional well-being through cognitive and behavioral skills development. However, this study extended the intervention to 12 weeks to allow for a deeper exploration of the growth mindset and resilience concepts, which are particularly relevant to the cultural and educational context of boarding school students in China. Additionally, this program incorporated mindfulness and reflective activities—elements that were not part of Galante et al.’s (2021) original intervention—to better align with the specific needs of this study population. The decision to extend the intervention beyond the typical 4-to 8-week duration seen in other positive psychology interventions (Bolier et al., 2013) was grounded in research suggesting that longer programs enhance the retention of core concepts while maintaining participant engagement. The intervention was carefully paced, ensuring that each component of the PERMA model—positive emotions, engagement, relationships, meaning, and accomplishment—was thoroughly covered. Each session began with mindfulness meditation, followed by theoretical instruction delivered via videos, practical activities such as the “Three Good Things” exercise, and concluded with reflective writing to reinforce the concepts introduced during the session.

This approach not only connects the theoretical underpinnings of the PERMA model with practical application but also adapts established positive psychology programs to suit the unique educational and cultural context of Chinese boarding school students. The structured weekly sessions ensured that students could effectively apply growth mindset strategies in both academic and personal contexts, ultimately supporting their emotional regulation and overall well-being.

### 3.5 Outcome measures

Two primary outcomes were measured in this study:

#### 3.5.1 Growth mindset

The Growth Mindset Scale (GMS), developed by Dweck (2006), measures participants’ belief in the potential to improve intelligence through effort. This 3-item scale uses a six-point Likert scale (1 = strongly agree, 6 = strongly disagree). In this study, Cronbach’s alpha for the GMS was 0.901, indicating good reliability.

#### 3.5.2 Resilience

The Connor-Davidson Resilience Scale (CD-RISC) (Connor and Davidson, 2003) is a 25-item scale that assesses an individual’s resilience in areas such as adaptability, control, and personal strength. Responses range from 1 (not true at all) to 5 (true nearly all of the time). In this study, the CD-RISC demonstrated good reliability, with a Cronbach’s alpha of 0.927.

### 3.6 Sample size calculation

Using G*Power 3.1.9.7 (Cohen, 1992), the required sample size was calculated with a significance level of 0.05 and a statistical power of 80%. A minimum of 128 participants was required. To account for potential nonresponse, the sample size was increased by 30% (Israel, 1992), resulting in a total of 167 adolescents participating in the study.

### 3.7 Data analysis

Statistical analyses were performed using SPSS version 27. Baseline comparisons between the experimental and control groups on demographic variables (age and gender) and outcome variables (growth mindset and resilience) were conducted using independent samples *t*-tests. Pre-test and post-test scores were compared using repeated-measures ANOVA to assess the impact of the intervention on growth mindset and resilience over time.

## 4 Results

### 4.1 Implementation results

All 167 participants completed the intervention without any missing data, achieving a 100% compliance rate throughout the study. The experimental group attended an average of 12 sessions, demonstrating strong engagement with the positive education program. This high level of participation ensured that the integrity of the data was maintained and that the results accurately reflect the intervention’s impact. The complete data set facilitated a robust analysis of the outcomes related to growth mindset and resilience.

### 4.2 Baseline sample characteristics

As shown in Table 1, the demographic characteristics of the participants (*N* = 167) were based on gender and age across experimental and control groups. In terms of gender, 83 participants were male (54.2% in the experimental group and 45.8% in the control group), while 84 were female (46.4% in the experimental group and 53.6% in the control group). The participants’ ages ranged from 12 to 16 years, with the majority being 14 years old (32.3%), followed by 13 years (28.1%), 15 years (23.4%), 12 years (12.0%), and the smallest group aged 16 years (4.2%). The sample was nearly equally distributed between the two groups.

**Table 1:** Demographic characteristics of participants according to gender and age (*N* = 167).

Variable	Experimental group	Control group	Total
Gender			
Male	45 (54.2%)	38 (45.8%)	83 (100.0%)
Female	39 (46.4%)	45 (53.6%)	84 (100.0%)
Total	84 (50.3%)	83 (49.7%)	167 (100.0%)
Age			
12 years old	11 (13.1%)	9 (10.8%)	20 (12.0%)
13 years old	23 (27.4%)	24 (28.9%)	47 (28.1%)
14 years old	27 (32.1%)	27 (32.5%)	54 (32.3%)
15 years old	20 (23.8%)	19 (22.9%)	39 (23.4%)
16 years old	3 (3.6%)	4 (4.8%)	7 (4.2%)
Total	84 (50.3%)	83 (49.7%)	167 (100.0%)

An independent samples *t*-test revealed no significant differences between the experimental and control groups in terms of resilience and growth mindset at baseline. As shown in Table 2, the mean score for growth mindset in the experimental group was *M* = 32.12, SD = 6.08, while the control group had a mean score of *M* = 34.14, SD = 5.61; *t*(165) = 0.21, *p* = 0.68. For resilience, the experimental group had a mean score of *M* = 52.12, SD = 12.79, and the control group had *M* = 54.67, SD = 12.78; *t*(165) = −0.46, *p* = 0.67. These results indicate that the two groups were homogenous at the start of the study, as presented in Table 2.

**Table 2:** The pre-test of experimental and control groups on growth mindset and resilience.

	Group	*N*	*M*	SD	SEM	*t*	*p*
Growth mindset	Experimental	84	32.12	6.08	0.98	0.21	0.68
	Control	83	34.14	5.61	0.87		
Resilience	Experimental	84	52.12	12.79	1.32	−0.46	0.67
	Control	83	54.67	12.78	1.27		

### 4.3 Descriptive statistics

Table 3 presents the descriptive statistics for the effectiveness of the positive education intervention on growth mindset and resilience over time. At baseline, the mean score for growth mindset was 2.71 (SD = 1.10), which increased to 3.46 (SD = 0.86) post-intervention. Similarly, the mean score for resilience increased from 3.26 (SD = 0.77) at baseline to 3.80 (SD = 0.62) post-intervention. These increases were statistically significant (*p* < 0.001), indicating that participants’ growth mindset and resilience improved following the positive education intervention.

**Table 3:** Descriptive statistics for the effectiveness of positive education intervention over time.

Item	*M*	SD	*p*
Growth mindset			<0.001
Baseline	2.71	1.10	
Post-intervention	3.46	0.86	
Resilience			<0.001
Baseline	3.26	0.77	
Post-intervention	3.80	0.62	

### 4.4 Analysis of variance

To assess the effectiveness of the intervention across the two time points (baseline and post-intervention), repeated-measures ANOVAs were conducted for both growth mindset and resilience (Table 4). The results showed a significant main effect of time on growth mindset, *F*(2, 211.07) = 65.36, *p* < 0.001, with a large effect size (multivariate eta squared = 0.96). For resilience, there was also a significant main effect of time, *F*(2, 160.57) = 33.19, *p* < 0.001, although the effect size was smaller (multivariate eta squared = 0.21).

**Table 4:** Summary of the repeated-measures ANOVAs for each scale.

Source of variation	SS	*df*	MS	*F*	Multivariate *η^2^*
Growth mindset	171.33	2	42.60	65.36^***^	0.96
Resilience	177.14	2	6.43	33.19^***^	0.21

SS, sum of squares; MS, mean square; df, degrees of freedom; ^***^ *p* < 0.001.

## 5 Discussion

This study contributes to the growing body of research on the effectiveness of positive education interventions in fostering adolescents’ personal growth and resilience, particularly within boarding schools. While previous studies have demonstrated the benefits of positive education in improving mental health outcomes—such as increased life satisfaction, hope, self-esteem, and reductions in depression, stress, and anxiety (Waters and Loton, 2019)—their specific impact in boarding school settings remains underexplored. By examining the effects of a 12-week positive education program, grounded in the PERMA model, on the growth mindset and resilience of adolescents in Chinese boarding schools, this study addresses this gap. These results support the conclusion that the intervention was effective in enhancing both resilience and growth mindset.

The significant improvements in both growth mindset and resilience suggest that the positive education intervention successfully contributed to psychological development in these areas. The large effect size for growth mindset indicates that the intervention had a particularly strong impact on participants’ attitudes toward learning and personal growth, while the moderate effect size for resilience suggests that participants became better equipped to cope with challenges. The results also highlight the potential for targeted interventions to significantly influence cognitive and emotional factors over a relatively short period.

Practically, this research offers valuable insights for educational policymakers and practitioners by highlighting the effectiveness of positive education interventions in fostering a growth mindset and resilience. The findings suggest that these interventions can serve as a proactive and effective alternative to traditional mental health education. By demonstrating the potential for positive education programs to enhance personal growth and resilience among students, this study supports the integration of well-being and resilience-building initiatives into boarding school curricula. This is especially important given the growing mental health challenges faced by adolescents, such as depression and anxiety. Moreover, the research emphasizes the need for culturally sensitive interventions that are tailored to meet the unique needs of different student populations, ensuring that positive education programs are both impactful and contextually relevant across diverse educational settings.

In detail, the findings demonstrate that positive education interventions led to a substantial enhancement in growth mindset among participants, contrasting with some previous studies. For instance, Saraff and Tiwari (2020) found that mindfulness interventions positively influenced growth mindset in college students, whereas Vuorinen et al. (2019) reported no significant effect of character strengths interventions on Finnish adolescents’ growth mindset. The pronounced improvement observed in this study may be attributed to the unique characteristics of the Chinese boarding school population and the specific components of the intervention, such as goal-setting exercises, which directly targeted mindset development. This highlights the importance of context-specific approaches in designing effective educational interventions that cater to the cultural and psychological needs of distinct populations.

This study supports the notion that positive education can foster a growth mindset by cultivating positive emotions, engagement, relationships, meaning, and achievement (Seligman, 2018) while simultaneously satisfying essential psychological needs. The findings not only align with theoretical predictions but also provide empirical evidence that positive education interventions can effectively enhance key psychological attributes, such as a growth mindset, which are essential for academic success and personal development in adolescents. Furthermore, these results confirm that positive education interventions significantly enhance resilience among boarding school adolescents. The improvement in resilience observed in this study aligns with findings from diverse cultural contexts, including Finland, Zambia, Iran, the U.S., and China (Felver et al., 2019; Vuorinen et al., 2019; Gabana et al., 2022; Seale et al., 2022; Shafiee Rad and Jafarpour, 2023). Notably, this research extends the generalizability of positive education interventions in enhancing resilience, particularly in a Chinese boarding school context. While previous research in China, such as Zhang et al. (2022), focused on adolescents with anxiety symptoms, this study targeted the general boarding school population, demonstrating that resilience-building interventions can be effective across a broader spectrum of students, not just those with preexisting mental health concerns.

The implications of this study are multifaceted. The positive outcomes suggest that boarding schools can benefit significantly from incorporating positive education interventions to foster a supportive and enriching educational environment. By promoting mental health and equipping students with essential psychological tools, these interventions can enhance both personal growth and academic success. Additionally, the study emphasizes the importance of culturally tailored interventions, which can lead to more effective outcomes by addressing the unique challenges and strengths of different student populations. Moreover, the robustness of positive education interventions in producing meaningful psychological changes within a relatively short intervention period of 12 weeks suggests they can be a cost-effective and efficient strategy for enhancing student well-being in educational settings. Future research should explore the long-term sustainability of these effects and evaluate their applicability across diverse cultural and educational contexts. Furthermore, investigating the mechanisms through which these interventions influence growth mindset and resilience, potentially through qualitative methodologies, can provide deeper insights into students’ experiences and perceptions.

In conclusion, this study provides compelling evidence for the efficacy of positive education interventions in promoting a growth mindset and resilience among Chinese boarding school adolescents. By demonstrating improvements in these key psychological constructs, the research validates the theoretical foundations of the PERMA model while offering practical recommendations for educational policy and practice. The integration of these interventions into boarding school curricula holds promise for enhancing the mental health and overall well-being of adolescents, fostering a more resilient and growth-oriented student population.

## 6 Limitations

While this study offers valuable insights, several limitations should be acknowledged. First, the geographical scope of the study was restricted to a specific region, focusing on boarding schools in China. Given the wide distribution of boarding schools across the country, the findings may not be fully generalizable to all boarding adolescents, particularly those in different socio-cultural or educational contexts. Second, the study design was limited by time constraints. Although the repeated measures design captured data at two time points, the absence of a delayed post-test follow-up limits the understanding of the long-term effects of the positive education intervention. Future studies should include additional time points, such as a follow-up after 2 or 3 months, to better assess the sustainability and longer-term impact of the intervention. Third, although the study is based on the ecological model of resilience, which emphasizes multiple levels of influence (e.g., individual, social, and community), the scale used in this study primarily evaluated individual traits of resilience. This limited focus does not fully account for other ecological factors such as family, school, or community influences, which are critical components of the resilience framework. Future research should incorporate broader measures that assess resilience across multiple ecological domains to provide a more comprehensive understanding of resilience-building interventions. Finally, it is important to note that only participants who completed both pre-and post-intervention measurements were included in the analysis. This inclusion criterion may limit the generalizability of the findings to a broader population of adolescents, as participants who dropped out may differ significantly from those who completed the intervention.

## 7 Conclusion

This study explores the impact of a positive education intervention on the growth mindset and resilience of Chinese boarding middle school students. The conclusions drawn from the results of this study can be summarized in three key aspects. First, the positive education intervention significantly influences both growth mindset and resilience among boarding middle school students. After the intervention, there is a notable increase in both growth mindset and resilience. Second, routine mental health education shows no significant impact on growth mindset and resilience. Third, routine mental health education is ineffective in promoting growth mindset and resilience, whereas the positive education intervention demonstrates significantly better results. These findings suggest that targeted interventions like the positive education intervention are crucial for fostering positive psychological development in educational contexts, highlighting the need for innovative approaches to support student resilience and growth mindset.

## Data Availability

The raw data supporting the conclusions of this article will be made available by the authors, without undue reservation.
